# The Role of Tobacco, Alcohol, and Obesity in Neoplastic Progression to Esophageal Adenocarcinoma: A Prospective Study of Barrett's Esophagus

**DOI:** 10.1371/journal.pone.0052192

**Published:** 2013-01-03

**Authors:** Sheetal Hardikar, Lynn Onstad, Patricia L. Blount, Robert D. Odze, Brian J. Reid, Thomas L. Vaughan

**Affiliations:** 1 Public Health Sciences Division, Fred Hutchinson Cancer Research Center, Seattle, Washington, United States of America; 2 Department of Epidemiology, University of Washington, Seattle, Washington, United States of America; 3 Human Biology Division, Fred Hutchinson Cancer Research Center, Seattle, Washington, United States of America; 4 Department of Medicine, University of Washington, Seattle, Washington, United States of America; 5 Department of Pathology, Harvard Medical School and Brigham and Women's Hospital, Boston, Massachusetts, United States of America; 6 Department of Genome Sciences, University of Washington, Seattle, Washington, United States of America; Vanderbilt University Medical Center, United States of America

## Abstract

**Background:**

Esophageal adenocarcinoma (EA) incidence in many developed countries has increased dramatically over four decades, while survival remains poor. Persons with Barrett's esophagus (BE), who experience substantially elevated EA risk, are typically followed in surveillance involving periodic endoscopy with biopsies, although few progress to EA. No medical, surgical or lifestyle interventions have been proven to safely lower EA risk.

**Design:**

We investigated whether smoking, obesity or alcohol could predict progression to EA in a prospective cohort of 411 BE patients. Data were collected during personal interview. Adjusted hazard ratios (HR) were estimated using Cox regression.

**Results:**

39% had body mass index (BMI) over 30 and 64% had smoked cigarettes. Main analyses focused on those with at least 5 months of follow-up (33,635 person-months), in whom 45 developed EA. Risk increased by 3% per year of age (trend p-value 0.02), with approximate doubling of risk among males. EA risk increased with smoking pack-years (trend p-value 0.04) and duration (p-value 0.05). Compared to never-smokers, the HR for those in the highest pack-year tertile was 2.29 (95%CI 1.04–5.07). No association was found with alcohol or BMI, whereas a suggestion of increased risk was observed in those with higher waist-hip ratio, especially among males.

**Conclusion:**

EA risk significantly increased with increasing age and cigarette exposure. Abdominal obesity, but not BMI, was associated with a modest increased risk. Continued follow-up of this and other cohorts is needed to precisely define these relationships so as to inform risk stratification and preventive interventions.

## Introduction

A rapid increase in incidence of and mortality from esophageal adenocarcinoma (EA) has been observed over the past four decades in the Western world [1]–[4]. Although the absolute incidence of EA varies dramatically by gender and race, few demographic groups have been spared from the increases. Moreover survival of persons with EA remains abysmal, with most succumbing to the disease within a year [5], [6]. Extensive research has identified the likely main causes of most EAs, implicating obesity, cigarette smoking, reflux and, to a lesser extent, diet as potentially modifiable etiologic factors [7]. Additionally, a growing number of observational studies suggest that aspirin and other non-steroidal anti-inflammatory drugs (NSAID) may be effective in reducing the risk of EA [8], [9], even in those who have high-grade dysplasia or significant genetic abnormalities [10].

Barrett's esophagus (BE), a columnar intestinal metaplasia of the lower end of the esophagus that develops in approximately 10–15% of patients with gastro-esophageal reflux, is a relatively easily-discerned precancerous condition associated with a 30-fold or higher increase in EA incidence [11]–[13]. BE can be found in most individuals with EA, implying that it is a precursor in neoplastic progression [11]. Consequently, extensive efforts have long been undertaken by clinicians to identify persons in the general population who have BE and enroll them into a long-term cancer surveillance program, in the hope of identifying early-stage esophageal cancers amenable to surgical cure. Some studies have shown that EA's diagnosed in such surveillance programs have earlier-stage disease and increased survival compared to EA's diagnosed outside of such programs [14]. Although the risk of EA among persons with BE is increased relative to the general population, the absolute risk of individuals with BE progressing to EA remains low, approximately 0.4–0.7% per year [15], [16]. It is not yet clear why only some persons with BE undergo neoplastic changes in their Barrett's segment and progress to cancer, while in most others it remains a relatively benign condition throughout their life.

Clinical and demographic factors that have shown some promise in being predictive of malignant transformation in BE are male gender [17], [18], increasing age [17], length of Barrett's segment [19]–[22], duration of BE [20], and size of hiatal hernia [21]. Decreased risk of progression has also been shown among aspirin and NSAID users [8], [9] and consumers of multivitamins, vitamin C, and vitamin E [23]. If individuals at high risk of neoplastic progression can be more accurately distinguished from those who are likely to follow a benign course (risk stratification), using host and lifestyle factors combined with validated markers of risk from serum and esophageal tissue, then substantial improvement in clinical management of BE could be achieved.

A number of population-based case-control studies and pooled analyses have examined the effect of smoking and obesity on EA development, [24], [25], but the stage(s) at which they act are largely unknown. While several studies have examined the associations between smoking, obesity and development of BE [26], [27], very few have looked at whether cigarette smoking and obesity promote development of EA among persons who already have BE. Moreover, some of these studies were limited by their number of EA cases, thus necessitating further evidence [20], [22], [28]. We sought to investigate the associations between measures of cigarette smoking, alcohol, and obesity and risk of progression to EA in a prospective cohort study of 411 patients with BE.

## Methods

### Study participants

This report is based on participants of the Seattle Barrett's Esophagus Study (SBES), a prospective cohort of BE patients originally established in 1983 [10], [29]. Each participant in this cohort undergoes periodic endoscopic surveillance with multiple biopsies of the Barrett's segment, as per a standard protocol [11], [30]. In 1995, the protocol was expanded to include an extensive personal interview, dietary and anthropometric assessments and collection of blood samples in addition to the periodic endoscopies with biopsies. Ongoing participants underwent this expanded evaluation at their first clinical visit on or after Feb 1, 1995; for the current report this is referred to as the baseline evaluation. At subsequent clinical visits, shorter personal interviews updated information collected at the baseline evaluation. This report is based on SBES participants enrolled for observation between February 1, 1995 and September 30, 2009. During this period, 427 participants with BE and no history of esophageal cancer were interviewed, of whom 411 (96.3%) had at least one follow-up visit and thus were eligible for analyses. This study was approved by the Institutional Review Boards at the University of Washington and Fred Hutchinson Cancer Research Center.

### Baseline exposure assessment

At the baseline evaluation, each participant underwent a structured personal interview conducted in person by trained staff. The interview lasted approximately 45 minutes and data regarding known and suspected risk factors for EA was collected. Information on participants' medical, family, and medication history (along with current medication use), tobacco use, beverage consumption, and diet was collected in addition to their demographics. At this time anthropometric measurements were taken by trained staff. The participants also provided fasting blood samples prior to the endoscopy. Follow-up assessments occurred at six month to two year intervals depending on the patients' risk of developing EA (based on histologic and flow cytometric assessments) with high-risk patients returning every six months [31]. These follow-up evaluations included endoscopy, a shorter interview with anthropometric measurements, and blood collection.

To collect data on smoking habits, participants were asked if they had smoked at least one cigarette/day for six months or longer (ever regular use), the intensity with which they smoked (number of cigarettes/day), duration for which they had been smoking and time since quitting. Cumulative pack-years of smoking were calculated based on the number of cigarette packs smoked per day and the number of years smoked. We calculated the number of alcoholic drinks consumed per day by combining participants' responses for beer, wine and liquor consumption. In models testing the association with alcohol, we used a categorical variable for total alcohol as well as for specific beverage intake. A history of aspirin and other NSAID use was collected at baseline interview, and updated at each follow-up visit as previously reported [10], [29].

Anthropometric measurements including height, weight, waist circumference and hip circumference were measured at baseline and at every follow-up visit using a standardized protocol. Waist circumference was measured at the waist at the level of the iliac crest. Hip circumference was measured at the largest circumference around the buttocks. Body mass index (BMI) was calculated as weight in kilograms divided by height in meters squared (kg/m^2^) and categorized into four pre-defined categories: ≤25, >25-≤30, >30-≤35 and >35. The waist and hip circumferences were used to calculate the waist-hip ratio (WHR) and categorized into sex-specific quartiles based on all participants.

### Ascertainment of end points

Biopsies taken during follow-up visits were used to assess the presence of EA. The methods for endoscopy and biopsy have been described previously [31]–[33]. Briefly, four-quadrant biopsies were obtained at 2 cm intervals from the Barrett's segment (1 cm intervals for those with history of high-grade dysplasia) and were fixed, processed and interpreted by a single pathologist blinded to the exposure status. Individuals with high grade dysplasia at their initial endoscopy (80/411 participants; 19.46%) were re-endoscoped twice more within 4 months so as to detect any occult cancers missed at baseline endoscopy. At each follow-up visit, participants were classified according to the maximum histological abnormality present (BE, low-grade dysplasia, high-grade dysplasia or EA). The study end-point for this report was development of EA, defined histologically as invasion of neoplastic epithelium beyond the basement membrane of the esophageal mucosa into the surrounding lamina propria, muscularis mucosa or submucosa [31].

### Statistical analysis

Hazard ratios (HRs) and 95% confidence intervals (CI) were estimated using Cox proportional hazards regression. Time to EA development was used as the underlying time metric with entry and exit times defined as the date of baseline visit endoscopy, and date of endoscopic cancer diagnosis or last follow-up, respectively.

We evaluated the association between anthropometric measures and EA by including them as continuous variables in the Cox models as well as by grouping participants into categories (BMI) or quartiles (WHR, waist, and hip circumferences) of the respective variables. Associations between cigarette smoking and EA were analyzed based on ever use, smoking duration, smoking intensity and cumulative exposure to smoking based on pack-years. In evaluation of these associations, never-smokers of cigarettes were used as a reference group and the associations were tested in tertiles of the various smoking-related variables. Alcohol-related analyses included association of EA with categorical variables for total alcohol intake as well as individual beverage types (beer, wine and liquor). All models were adjusted for age, gender, and NSAID use. The anthropometry-related models were also adjusted for smoking status; the tobacco-related models were adjusted for baseline WHR and the alcohol-related models were adjusted for smoking and baseline WHR. Tests for trend were based on the likelihood-ratio test associated with addition of the variable under consideration in its continuous form. Effect modification was examined through stratified analyses and the inclusion of interaction terms in regression models. All p-values presented in this paper are two-tailed and p-values less than 0.05 were considered statistically significant. The proportional hazards assumption was confirmed by computing interaction terms for covariates with time and testing them for statistical significance at the 0.05 level. Analyses were performed with the STATA statistical package, release 11.

## Results

During the study period, 427 participants with BE were enrolled into the SBES cohort; of these 16 did not yet have any follow-up visits and were excluded from the analyses. Participant characteristics for the remaining 411 persons are shown in Tables 1 and 2. Consistent with demographics of the disease, Caucasians (96.6%) and males (81.3%) made up the majority of the cohort. The percentage of males in the cohort was slightly higher than reported in other BE studies [34]. The mean age of the cohort was 61.2 years. A total of 39.2% of the participants had a BMI greater than 30 kg/m^2^, with 8.8% having a BMI over 35 kg/m^2^. Most were current or former cigarette smokers (64%), reported having regularly used alcohol in their lifetime (81.5%), and had regularly taken NSAID at some point in their life (60.6%).

**Table 1:** Selected characteristics of all participants in the SBES cohort at baseline.

	Total (n = 411)		Males (n = 334)		Females (n = 77)	
	Number	%	Number	%	Number	%
**Age (years)**						
30–44.9	30	7.3	25	7.5	5	6.5
45–54.9	97	23.6	83	24.9	14	18.2
55–64.9	110	26.8	84	25.2	26	33.8
65–74.9	114	27.7	93	27.8	21	27.3
≥75	60	14.6	49	14.7	11	14.3
**Race**						
White	397	96.6	324	97.0	73	94.8
Non-white	14	3.4	10	3.0	4	5.2
**BMI (kg/m^2^)**						
≤25	56	13.6	41	12.3	15	19.5
25.1-≤30	194	47.2	167	50.0	27	35.1
30.1-≤35	125	30.4	102	30.5	23	29.9
>35	36	8.8	24	7.2	12	15.6
**Cigarette smoking**						
Current	40	9.7	28	8.4	12	15.6
Former	223	54.3	192	57.5	31	40.3
Never	148	36.0	114	34.1	34	44.1
**NSAID use** †						
Current	169	41.1	145	43.4	24	31.2
Former	79	19.2	58	17.4	21	27.3
Never	162	39.4	130	38.9	32	41.6
**Alcohol (drinks/day)**						
0	76	18.5	52	15.6	24	31.2
0.01–1.00	134	32.6	102	30.5	32	41.6
1.01–2.99	93	22.6	80	24.0	13	16.9
≥3.00	108	26.3	100	29.9	8	10.4

† Current NSAID use history for one male participant was missing.

**Table 2:** Anthropometric measurements of all participants in the SBES cohort at baseline.

		Entire cohort		Males		Females	
		(n = 411)		(n = 335)		(n = 76)	
		No.	Mean	No.	Mean	No.	Mean
**Waist-Hip ratio** †	Q1	102	0.86	83	0.90	19	0.78
	Q2	102	0.93	83	0.95	19	0.84
	Q3	102	0.97	83	0.98	19	0.89
	Q4	103	1.02	83	1.03	20	0.96
**Waist circumference** †	Q1	102	34.42	83	35.78	19	30.48
	Q2	102	38.42	82	38.75	19	35.34
	Q3	100	40.83	84	41.19	19	39.43
	Q4	105	45.17	83	45.50	20	43.63
**Hip circumference** †	Q1	102	37.98	82	38.24	17	36.57
	Q2	102	40.58	84	40.57	21	40.39
	Q3	102	42.66	83	42.54	19	43.85
	Q4	103	46.85	83	45.86	20	50.38

† Two males had missing waist and hip circumferences at baseline.

Q1: Quartile 1, Q2: Quartile 2, Q3: Quartile 3, Q4: Quartile 4.

Waist-Hip ratio- Entire cohort: Q1 0.72-, Q2 0.91-, Q3 0.95-, Q4 0.99-; Males: Q1 0.72-, Q2 0.93-, Q3 0.96-, Q4 1.00-; Females: Q1 0.72-, Q2 0.81-, Q3 0.87-, Q4 0.91-.

Waist circumference – Entire chort: Q1 25.5-, Q2 37.1-, Q3 39.6-, Q4 42.5-; Males: Q1 25.5-, Q2 37.6-, Q3 39.8-, Q4 42.6-; Females: Q1 25.5-, Q2 33.5-, Q3 37.9-, Q4 40.8-.

Hip circumference - Entire chort: Q1 32.0-, Q2 39.5-, Q3 41.7-, Q4 43.9-; Males: Q1 32.0-, Q2 39.6-, Q3 41.7-, Q4 43.4-; Females: Q1 32.0-, Q2 39.0-, Q3 41.8-, Q4 40.8-.

Table 2 summarizes the distribution of anthropometric measurements overall and by gender. The mean WHR for the entire cohort was 0.95 (males 0.96, females 0.87). These data are in accordance with the known patterns of obesity among men and women: men had higher waist and abdominal circumferences pointing towards central adiposity while females had higher hip and thigh circumferences. The correlation between baseline BMI and WHR was relatively weak (r = 0.20; p-value = 0.001) suggesting that these parameters could be jointly evaluated with respect to their EA risk.

Fourteen persons had less than five months of follow-up, during which 11 were diagnosed with cancer. These 14 participants were excluded from the main statistical analyses (Tables 3,4,5) due to an *a priori* concern that cancers found during this early period of intensive search for an occult cancer may have been present at baseline. Analyses including all 411 participants were also conducted and results are noted where different. The 397 participants accumulated 33,635 person-months (2802.9 person-years; median 6.2 years, interquartile range 2.6–11.4 years) of follow-up during which 45 developed EA.

**Table 3:** Adjusted hazard ratios (HRs) and 95% confidence intervals (CIs) of esophageal adenocarcinoma among cigarette smokers in the SBES cohort.

	EA/total	Unadjusted HR(95% CI)	Adjusted for age† and gender HR(95% CI)	Adjusted for age†, gender, WHR‡ and NSAID use¶ HR(95% CI)
**Ever use**				
Never	11/144	REF	REF	REF
Ever	34/253	1.87 (0.95–3.69)	1.72 (0.87–3.41)	1.57 (0.78–3.14)
**Duration (years)**				
Nonsmokers	11/144	REF	REF	REF
T1 (0.5-<17)	9/83	1.46 (0.60–3.53)	1.49 (0.61–3.60)	1.41 (0.58–3.42)
T2 (17-<31)	10/82	1.54 (0.66–3.64)	1.43 (0.61–3.39)	1.30 (0.54–3.11)
T3 (31+)	15/88	2.71 (1.24–5.91)	2.25 (1.02–4.94)	2.00 (0.89–4.46)
*p-value trend* *		*0.003*	*0.03*	*0.05*
**Intensity**				
Nonsmokers	11/144	REF	REF	REF
<1 pack/day	8/87	1.18 (0.47–2.93)	1.16 (0.47–2.88)	1.04 (0.41–2.59)
1 pack/day	13/71	2.64 (1.18–5.90)	2.28 (1.02–5.13)	2.17 (0.96–4.95)
>1 pack/day	13/95	2.01 (0.90–4.49)	1.84 (0.82–4.13)	1.68 (0.74–3.82)
*p-value trend* *		*0.02*	*0.07*	*0.10*
**Cumulative exposure (pack-years)**				
Nonsmokers	11/144	REF	REF	REF
T1 (0.05-<14)	7/84	1.07 (0.41–2.75)	1.08 (0.42–2.80)	1.04 (0.40–2.69)
T2 (14-<36)	11/84	1.75 (0.76–4.03)	1.64 (0.71–3.80)	1.43 (0.61–3.37)
T3 (36+)	16/85	3.02 (1.40–6.50)	2.48 (1.14–5.42)	2.29 (1.04–5.07)
*p-value trend* *		*0.002*	*0.02*	*0.04*

† – Age modeled as a continuous variable,

‡ – WHR modeled as a continuous variable,

¶ – NSAID use modeled as a categorical variable (Current, Former, never).

T1: Tertile 1, T2: Tertile 2, T3: Tertile 3.

* Test for trend was based on the likelihood-ratio test associated with addition of the variable under consideration in its continuous form.

**Table 4:** Adjusted hazard ratios (HRs) and 95% confidence intervals (CIs) of esophageal adenocarcinoma for alcohol intake in the SBES cohort.

	EA/Total	Unadjusted HR(95% CI)	Adjusted for age†, gender, WHR‡ and NSAID use¶ HR(95% CI)	Adjusted for age†, gender, WHR‡, NSAID use¶ and cigarette smoking€ HR(95% CI)
**Total alcohol (drinks/day)**				
0	8/75	REF	REF	REF
>0–1	9/135	0.63 (0.24–1.62)	0.65 (0.25–1.68)	0.56 (0.21–1.50)
>1–3	16/102	1.47 (0.63–3.44)	1.43 (0.60–3.45)	1.24 (0.50–3.08)
>3	12/85	1.35 (0.55–3.30)	1.23 (0.48–3.14)	1.00 (0.37–2.69)
*p-value trend* *		*0.770*	*0.941*	*0.800*
**Beer (drinks/day)**				
0	12/143	REF	REF	REF
>0–1	21/171	1.57 (0.77–3.18)	1.61 (0.76–3.43)	1.43 (0.65–3.17)
>1–3	8/47	1.97 (0.80–4.81)	1.79 (0.69–4.61)	1.57 (0.59–4.19)
>3	4/33	1.53 (0.49–4.76)	1.53 (0.46–5.05)	1.34 (0.39–4.57)
*p-value trend* *		*0.679*	*0.877*	*0.941*
**Wine (drinks/day)**				
0	27/214	REF	REF	REF
>0–1	16/163	0.75 (0.40–1.39)	0.70 (0.37–1.30)	0.68 (0.37–1.27)
>1–3	2/16	1.33 (0.32–5.61)	1.19 (0.28–5.02)	1.35 (0.32–5.74)
>3	0/2	-	-	-
*p-value trend* *		*0.372*	*0.100*	*0.101*
**Liquor (drinks/day)**				
0	11/138	REF	REF	REF
>0–1	21/185	1.41 (0.68–2.93)	1.29 (0.62–2.71)	1.17 (0.54–2.53)
>1–3	9/45	3.06 (1.27–7.39)	2.86 (1.17–7.01)	2.51 (0.99–6.38)
>3	4/24	1.83 (0.58–5.74)	1.40 (0.42–4.62)	1.27 (0.38–4.27)
*p-value trend* *		*0.107*	*0.310*	*0.417*

† – Age as a continuous variable,

‡ –WHR as a continuous variable,

¶ – NSAID use as a categorical variable (Current, Former, never),

€ – Smoking as a categorical variable (Ever, never).

* Test for trend was based on the likelihood-ratio test associated with addition of the variable under consideration in its continuous form.

**Table 5:** Adjusted hazard ratios (HRs) and 95% confidence intervals (CIs) of esophageal adenocarcinoma (EA) for BMI and waist-hip ratio (WHR) in the SBES cohort.

	EA/total	Unadjusted HR(95% CI)	Adjusted for age† and gender HR(95% CI)	Adjusted for age†, gender, cigarette smoking‡ and NSAID¶ use HR(95% CI)
**BMI**				
BMI continuous	45/397	0.99 (0.93–1.07)	1.02 (0.95–1.10)	1.01 (0.94–1.10)
BMI categories				
≤25	6/54	REF	REF	REF
>25-≤30	22/188	0.91 (0.37–2.24)	1.00 (0.40–2.49)	0.88 (0.35–2.19)
>30-≤35	13/120	0.84 (0.32–2.22)	0.99 (0.37–2.63)	0.81 (0.31–2.18)
>35	4/35	0.86 (0.24–3.05)	1.33 (0.36–4.89)	1.21 (0.32–4.48)
*p-value trend* *		*0.85*	*0.61*	*0.73*
**WHR**				
WHR male & female				
Q1	8/99	REF	REF	REF
Q2	12/99	1.67 (0.68–4.09)	1.58 (0.64–3.88)	1.38 (0.56–3.40)
Q3	12/97	1.46 (0.60–3.58)	1.40 (0.57–3.44)	1.24 (0.50–3.05)
Q4	13/100	1.88 (0.78–4.55)	1.61 (0.66–3.92)	1.48 (0.60–3.61)
*p-value trend* *		*0.01*	*0.12*	*0.16*
Q2–Q4	37/294	1.66 (0.77–3.56)	1.53 (0.71–3.29)	1.36 (0.63–2.94)
WHR male				
Q1	7/82	REF	REF	REF
Q2	11/80	1.84 (0.71–4.75)	1.67 (0.65–4.34)	1.44 (0.55–3.76)
Q3	11/78	1.62 (0.63–4.19)	1.50 (0.58–3.88)	1.32 (0.51–3.44)
Q4	12/82	1.98 (0.78–5.04)	1.72 (0.67–4.40)	1.53 (0.59–3.96)
*p-value trend* *		*0.04*	*0.08*	*0.12*
Q2–Q4	34/238	1.81 (0.80–4.08)	1.63 (0.72–3.69)	1.42 (0.62–3.26)
WHR female				
Q1	1/17	REF	REF	REF
Q2	1/19	0.87 (0.05–13.94)	1.03 (0.06–16.77)	3.01 (0.09–99.07)
Q3	1/19	0.71 (0.04–11.39)	0.77 (0.05–12.45)	0.82 (0.05–13.62)
Q4	1/18	1.21 (0.07–19.69)	0.91 (0.05–15.19)	0.95 (0.05–18.92)
*p-value trend* *		*0.97*	*0.86*	*0.75*
Q2–Q4	3/56	0.89 (0.09 –8.54)	0.89 (0.09–8.59)	1.04 (0.10–10.96)

† – Age modeled as a continuous variable,

‡ – Cigarette smoking modeled as a categorical variable (Current, Former, Never),

¶ – NSAID use modeled as a categorical variable (Current, Former, Never).

Q1: Quartile 1, Q2: Quartile 2, Q3: Quartile 3, Q4: Quartile 4.

* Test for trend was based on the likelihood-ratio test associated with addition of the variable under consideration in its continuous form.

The risk of progression to EA increased significantly with age (HR = 1.03 per year; 95%CI 1.00–1.06; p-trend = 0.02) and this association remained statistically significant after controlling for gender (p-trend = 0.014). Males were found to be at a non-significantly higher risk for EA development than females (HR = 2.32; 95%CI 0.83–6.49).

The association between various cigarette smoking-related measures and EA is described in Table 3. Smoking-related parameters were computed on the basis of the participants' responses at the baseline visit. Ever smokers of cigarettes were at approximately 90% (HR = 1.87, 95%CI 0.95–3.69) higher risk of EA in univariate analyses. This association was attenuated somewhat after control for the confounding effects of age, gender, WHR and NSAID use (HR = 1.57, 95%CI 0.78–3.14). Fully-adjusted analyses which examined dose-response relationships revealed statistically-significant trends with smoking duration (p_trend_ = 0.05) and cumulative exposure (p_trend_ = 0.04), but not with a summary measure of intensity (p_trend_ = 0.10). As compared to never-smokers, the HR for those in the highest tertile for pack-years of cigarette smoking was 2.29 (95%CI 1.04–5.07). We found no evidence that cigarette smoking cessation prior to baseline evaluation was associated with decreased risk (p_trend_ = 0.97). Neither WHR nor age substantially modified the relationship between smoking and EA risk (Data not shown).

Table 4 presents results regarding alcohol consumption and EA risk. In a model adjusted for age, gender, WHR, cigarette smoking and NSAID use, we found no association between drinking three or more alcoholic drinks per day and EA risk (HR = 1.00; 95%CI 0.37–2.69; p_trend_ = 0.80). We further examined this association by beverage type and found no evidence of increasing EA risk with increasing intake of beer or hard liquor. Although wine intake of up to one drink/day tended to decrease the risk associated with EA development, this decrease in risk was not statistically significant in univariate or adjusted models. Main analyses involving smoking and alcohol intake were repeated on the entire cohort of 411 persons (i.e. after including the 14 participants with 5 months or less of follow-up) but the overall results remained the same.

In analyses of anthropometry (Table 5), three sets of models were examined: unadjusted, adjusted for age and gender, and adjusted for age, gender, cigarette smoking and NSAID use. BMI was not associated with an increased EA risk in any of the analyses, whether modeled as a continuous or categorical variable. In addition, no substantial gender differences in the BMI-EA association were observed (Data not shown). We observed a significant trend (p_trend_ = 0.01) between increasing WHR and EA risk in univariate analyses, but adjustment for the confounding effects of age, gender, smoking and NSAID attenuated the association substantially such that the association was no longer statistically significant (p_trend_ = 0.16) (HR = 1.48; 95%CI 0.60–3.61, comparing extreme quartiles). Each of the four confounding variables contributed somewhat to the attenuation in HR, with no one variable predominating. The suggestive increased risk in the adjusted models was observed only among males (HR = 1.53; 95%CI 0.59–3.96; p_trend_ = 0.12) but not in females (HR = 0.95; 95%CI 0.05–18.92). Similar results as with WHR were obtained when waist and abdominal circumferences were evaluated for their relationship with EA, with an indication of a modest non-statistically significant increase in risk for the uppermost quartile (Data not shown). As with WHR, the increased risk of EA for both waist and abdominal circumferences was observed only among males, with no association in females. There was no suggestion of an association between EA risk and hip circumference either in the entire cohort or when the genders were evaluated separately (Data not shown).

We conducted exploratory analyses to examine whether the observed association with WHR among males varied by smoking status or age of the participants. In models adjusted for age and NSAID use, the elevated risk associated with men in the highest WHR quartile was only apparent among never smokers of cigarettes (Adjusted HR = 6.17; 95%CI 0.61–62.43; p_trend_ = 0.20) and not among regular cigarette smokers (Adjusted HR = 1.10; 95%CI 0.39–3.12; p_trend_ = 0.26). Additionally, the increase in EA risk associated with highest WHR quartile was greater among men under 61.5 years (Adjusted HR = 3.18; 95%CI 0.56–17.90; p_trend_ = 0.26) than among men over 61.5 years (Adjusted HR 1.24; 95%CI 0.40–3.79; p_trend_ = 0.21). While suggestive, these differences observed in the associations between WHR and EA by smoking status and age were not statistically significant and should be interpreted with caution due to the limited sample sizes involved (p_interaction_ = 0.50 and 0.34, respectively).

Of the 397 participants included in the above analyses, 17 (4.3%) received interventions such as mucosal ablation or endoscopic mucosal resection, an endoscopic therapy for the treatment of dysplastic changes in BE, prior to the end of their follow-up. To ensure that such interventions did not bias the reported HRs, we repeated the above analyses (Tables 3,4,5) after excluding people who underwent such treatments. The results for the remaining participants did not differ in important ways from the associations observed in the main statistical analysis (Data not shown). Of the original 411 participants, 103 (24.3%) individuals were already under surveillance before the start of the epidemiologic aspects of the study in 1995. To examine whether these participants, who may have had BE for a longer period of time than those participants newly entering the surveillance program, differed with respect to the role of obesity and cigarette smoking, we conducted sensitivity analyses dropping these 103 individuals and repeating statistical analyses in Tables 3,4,5. We found that while the associations with WHR were slightly stronger and those with cigarette smoking weaker in comparison to the main statistical analysis, none of the differences were statistically significant and the overall conclusions remained the same.

## Discussion

This prospective cohort study is one of the first to examine the independent and joint associations between cigarette smoking, alcohol, BMI and central adiposity and risk of neoplastic progression among persons diagnosed with BE. We observed a statistically-significant dose-response relationship with pack-years of smoking and smoking duration, but no apparent beneficial effect of smoking cessation. No evidence was found that alcohol consumption increased EA risk, when tested as total alcohol intake or by individual beverage type. We also found no indication that BMI was predictive of EA development. Rather, we found suggestive evidence that measures of central (abdominal) obesity, including WHR, waist and abdominal circumference, may modestly increase EA risk. The non-statistically significant increases we observed were stronger among males.

Cigarette smoking has long been known to be a major risk factor for esophageal squamous cell carcinoma; early reports examining the histology-specific relative risk estimates indicated a significant, but more modest role for smoking in EA [24]. Subsequent population-based case-control and cohort studies and a large pooled analysis based in the Barrett's and Esophageal Adenocarcinoma Consortium (BEACON) have since confirmed an approximately two-fold increase in risk among ever-smokers, with a strong and significant dose-response relationship with cumulative exposure, yielding a relative risk estimate of 2.7 (95%CI 2.2–3.4) among those with 45 or more pack-years of smoking [25].

The above studies examine the overall effect of smoking on EA development. Several case-control studies have also examined the association between smoking and development of BE, representing an intermediate stage in EA development [26], [27], [35], [36]. Three of these studies have observed statistically significant increases in risk among those in the highest cumulative exposure (pack-years) category, ranging from 1.7 to 2.5 [26], [27], [36] whereas a fourth [35] observed a more modest and non-significant 30% increased risk.

Our results, which indicate that high cumulative smoking exposure also raises risk of neoplastic progression from BE to EA (adjusted HR = 2.3), begin to fill in some gaps with regard to the overall role of smoking in EA development and the stages at which it acts among those already diagnosed with BE. Specifically, it appears that cigarette smoking likely plays roles of similar magnitude in both the development of BE and progression from BE to EA. This observation is in line with a recent study by Coleman *et al.* that reported a 2-fold increase in EA risk associated with current smoking among patients with BE [28]. The frequent observation that smoking cessation reduces EA risk only modestly and after many years is also consistent with a role of smoking in BE development, which can occur decades before EA diagnosis [25], [37]. The specific mechanisms by which smoking increases risk of both development of BE and progression from BE to EA are unknown, but may be a combination of exposure to chemicals such as N-nitrosoamines [38], [39], the promotion of GERD through the relaxing effects of tobacco smoke on the lower esophageal sphincter [40], and the continued inflammatory effects of smoking which promote cellular proliferation [7].

As with smoking, alcohol consumption is a strong established risk factor for esophageal squamous cell carcinoma [41], [42]. In contrast, there is little evidence to suggest that total alcohol consumption, or specific alcoholic beverages, modifies risk of EA in the general population. A pooled analysis of 1,821 EA cases and 10,854 controls in BEACON revealed an odds ratio of 0.97 (95%CI 0.68–1.36) among the heaviest drinkers, in marked contrast to the association with esophageal squamous cell carcinoma (odds ratio = 9.62; 95%CI 4.26–21.71) observed in the same analysis [43]. Fewer studies have examined the relationship between alcohol intake and risk of BE in the general population, but these also suggest no relationship [44]–[46]. There is little previous data examining the possible role of alcohol in the progression from BE to EA. However, our study, in combination with the large pooled analysis of EA risk in population-based studies, indicates that alcohol intake should not be a target for prevention activities.

The important role that obesity plays in EA development was recently summarized by Lagergren *et al.* [47], and has been confirmed in numerous population-based case-control and cohort studies [7], [24], [35], [48]–[54]. Case-control studies generally rely on recalled height and body-weight at various points in participants' lives, since anthropometric measurements after cancer diagnosis will often be modified by the cancer's wasting effects; therefore they cannot address the relative importance of weight (or BMI) and central adiposity. One cohort study with multiple pre-cancer measurements available found similar increased risk for BMI, WHR, waist circumference and fat mass [51]. However this study was limited by the small number of cancers observed, necessitating the combination of gastric cardia (n = 19) and EA (n = 11) in the analyses. Another cohort study had pant/skirt size available as proxy measures for waist circumference. Both BMI and pant/skirt size were significantly associated with risk of EA when analyzed separately; models adjusted for both factors suggested that BMI was more important although neither of the trend tests were significant [46]. Similarly, results from the EPIC cohort suggest that both BMI and abdominal obesity are important in EA development [52]. Case-control studies of BE as an outcome generally indicate that measures of abdominal obesity outweigh BMI in terms of strength of association with BE [26], [55]. Similar results were observed in a nested case-control study within a large cohort with pre-diagnostic measures of abdominal diameter [56], and a small clinical study where visceral fat was measured using computerized tomography [57].

Thus the relative roles of increased weight *per se* and abdominal obesity remain unclear. Overall, previous studies suggest that the effects of abdominal obesity are relatively strong with regard to the development of BE, and predominate over BMI. The present study suggests that, with regard to neoplastic progression to EA in persons with BE, the effects of abdominal obesity also predominate over BMI, but are more modest in their strength. There are several potential mechanisms which may be involved. First, deposition of abdominal fat may lead to increased intra-abdominal pressure which may directly cause gastro-esophageal reflux, a well-established risk factor for BE and EA [7], [58]. Second, adipose tissue is metabolically active, secreting pro-inflammatory cytokines and adipokines, which can promote cellular proliferation, reduce apoptosis, and trigger neoplastic transformation in the esophageal epithelium [7], [59], [60]. Results from the present report are consistent with a previous case-control study that suggested the relationship between obesity and EA may depend on the smoking status and age of the individual, with increased association between EA risk and obesity among non-smokers and younger individuals [61]. It is notable that the gender differences in EA risk observed in this study are consistent with the known demographics of the disease and can partly be explained by the characteristic abdominal distribution of fat seen in males as well as increased prevalence of smoking among males.

Our study has several strengths, the most important one being its prospective design. Additionally, misclassification of exposure status was minimized by measurement of central adiposity by trained staff, in contrast to use of self-reported data limited to BMI in some previous studies. Comprehensive information on various parameters of smoking and alcohol consumption before occurrence of EA enabled us to characterize these behaviors adequately and limit confounding. In main analyses, we also excluded cases of EA that occurred within the first 5 months of follow-up, thus minimizing potential bias by reverse causation, contrary to some previous studies that were unable to account for this bias [52].

While substantially larger than other well-characterized prospective cohort studies with anthropometric measures, the relatively small number of incident cases is an important limitation, leading to imprecise estimates, especially for the evaluation of effect modification. Additionally, we will not have captured all the incident cases of EA that occurred among our participants as follow-up ended with cessation of active surveillance. As some of the cohort members were being followed prior to 1995 (baseline evaluation for the purposes of this manuscript), our analyses included a mix of prevalent and incident cases of BE. This is a limitation of most studies of BE, as BE is an endoscopic diagnosis and the exact date of disease is rarely known. Another potential limitation is the possibility of residual confounding due to measured or unmeasured risk factors. In particular, we lacked data on *Helicobacter pylori* status, which could possibly confound the WHR-EA association. Not only has *H.pylori* been shown to decrease body weight by suppressing appetite, but it is also been observed in multiple population-based studies to be inversely related to risk of EA [62]–[64]. Finally, as our participants are from a specialized and relatively high-risk cohort of persons with histologically confirmed BE, the results presented in this report cannot necessarily be generalized to persons with BE in the general population or considered to be representative of the natural history of EA. Rather these estimates should be interpreted as describing the risk of progression to EA once diagnosed with BE.

In summary, we observed a statistically significant dose-response relationship with cigarette smoking pack-years as well as duration. We did not observe any association with alcohol intake or BMI. We also found that abdominal obesity, measured as WHR, may be modestly useful in predicting neoplastic progression to EA among Barrett's patients, especially among males. Further epidemiological studies with larger number of EA cases are required to validate these findings, especially among subgroups of people with BE in order to better understand the mechanisms by which tobacco and perhaps obesity increase the risk of neoplastic progression among Barrett's esophagus patients and their potential role in EA prevention.
